# Designing Self-Directed Learning Content for Medical Students: An AI-Driven Evaluation of Learning Outcomes

**DOI:** 10.7759/cureus.112519

**Published:** 2026-07-12

**Authors:** Roshni Agarwal, Amit Kumar, Vaibhav Agarwal, Leeza M Brahma, Shailja

**Affiliations:** 1 Microbiology, Autonomous State Medical College, Kanpur Dehat, IND; 2 Pathology, Regency Hospital Ltd., Kanpur, IND

**Keywords:** artificial intelligence, artificial intelligence and education, human-in-the-loop, medical education, self-directed learning (sdl), undergraduate and graduate medical education

## Abstract

Background: The National Medical Commission of India requires self-directed learning (SDL) in the MBBS curriculum. SDL is a fundamental component of competency-based medical education (CBME). Although generative artificial intelligence (AI) technologies such as ChatGPT (OpenAI, San Francisco, CA) and Gemini (Google, Mountain View, CA) have expanded options for improving SDL, concerns about accuracy, academic integrity, and overreliance persist. This study assessed the quality, effectiveness, and learning outcomes of undergraduate medical students in relation to an organized AI-assisted SDL process.

Material and methods: Eighty-three second-year MBBS students from the Department of Microbiology at the Autonomous State Medical College in Kanpur Dehat participated in a single-center pre-post interventional trial using a within-subject crossover design. After structured instruction in prompt engineering, AI verification techniques, and a human-in-the-loop workflow, participants completed a standard SDL assignment, followed by an AI-assisted SDL assignment. A validated 10-domain rubric (maximum score: 20) covering accuracy, relevance, completeness, organization, clarity, clinical examples, visuals, critical thinking, appropriateness, and originality was used to evaluate assignment quality. Pre- and post-tests measured knowledge acquisition, and student opinions and task completion times were also recorded. Statistical analyses included Cohen's d effect sizes, paired t-tests, and the intraclass correlation coefficient (ICC) for inter-rater reliability.

Result: In every rubric domain, AI-assisted SDL performed significantly better than traditional SDL (p < 0.001). Mean rubric scores improved by 29.1%, from 57.1% to 86.2% (Cohen's d = 2.8). Organization (+71.3%), completeness (+63.5%), and clarity (+62.5%) showed the largest gains. Knowledge scores increased from 5.2 to 8.8 out of 10 (p < 0.001; d = 2.5). Average task completion time decreased by 43%, from 44.5 to 25.4 minutes (p < 0.001; d = 1.5). Inter-rater reliability was excellent (ICC = 0.94). Most students reported feeling more confident (n = 65, 78%), synthesizing information more quickly (n = 76, 92%), and routinely using textbooks to verify AI-generated content (n = 71, 85%). The most commonly reported challenges were concerns about accuracy and timely formulation.

Conclusion: The systematic integration of generative AI into SDL significantly enhances MBBS students' learning efficiency, knowledge acquisition, and content quality. While highlighting the importance of maintaining human oversight to ensure accurate and responsible use of AI in medical education, our findings support integrating AI literacy and verification competencies into the medical curriculum.

## Introduction

The realm of contemporary medical education is undergoing a significant transformation, shifting from conventional, instructor-focused didactic models to more student-centered competency-based medical education (CBME) frameworks [1]. This movement centers on self-directed learning (SDL), a pedagogical method in which students assume primary responsibility for identifying their learning needs, establishing precise objectives, sourcing relevant materials, and critically assessing their progress [2]. In India, the National Medical Commission (NMC) has designated SDL as an essential element of the MBBS curriculum, acknowledging that the swift growth of medical knowledge requires future physicians to be lifelong learners capable of autonomously navigating extensive clinical information [3].

The advent of generative artificial intelligence (GenAI), especially large language models (LLMs) like ChatGPT (OpenAI, San Francisco, CA) and Gemini (Google, Mountain View, CA), signifies a potential "disruptive innovation" in this domain. These technologies could markedly improve SDL by offering tailored resources and immediate access to information, thereby assisting medical students in their pursuit of knowledge [4]. The advancement of these AI tools may transform the provision and experience of medical education, cultivating a more adaptable and knowledgeable healthcare staff [5]. These algorithms can summarize extensive data, produce organized notes, and develop intricate case vignettes with near-human linguistic proficiency. In medical education, AI serves as a "cognitive scaffold," assisting learners with lower-order activities such as drafting and organizing content, which ideally enables them to concentrate more on synthesis and verification [6].

The incorporation of AI into medical training presents considerable hurdles. "Hallucinations," where AI models provide factually inaccurate yet seemingly credible material, pose a significant threat to patient safety if incorporated into a student's knowledge base without careful examination [7]. Moreover, apprehensions about academic integrity, the deterioration of fundamental clinical reasoning abilities, and the opaque nature of AI decision-making have instilled a sense of reluctance among educators [8].

Notwithstanding these apprehensions, student utilization of AI technologies is already prevalent, frequently taking place in an "underground" or unregulated fashion. There is an immediate need for empirical investigation to ascertain whether organized, guided AI integration, referred to as the "Human-in-the-Loop" approach, can enhance learning outcomes in comparison to conventional methods [9]. This study seeks to bridge this gap by investigating the efficacy of a structured AI-driven workflow for improving the quality of SDL content generated by second-year MBBS students while simultaneously evaluating its effects on efficiency and learner confidence.

## Materials and methods

The study was conducted in the Department of Microbiology at the Autonomous State Medical College, Kanpur Dehat. The study comprised 83 second-year MBBS students from the 2024-25 academic cohort. This study aimed to investigate the efficacy of AI-integrated SDL content in improving the learning outcomes of medical students. The main aim was to ascertain if AI-assisted SDL enhances student performance relative to conventional SDL. This study was designed as a single-center, prospective within-subject pre-post study. In this design, each participant served as their own control by completing first a traditional SDL assignment (Assignment 1) and then an AI-assisted SDL assignment (Assignment 2). Eligible participants comprised students currently enrolled in the academic session who provided consent to participate, were available for both SDL sessions and pre- and post-tests, and possessed fundamental computer literacy and access to an internet-enabled device. Students were disqualified if they possessed prior formal experience in instructional design or AI-assisted learning; did not complete any aspect of the study, including assignments or assessments; or opted out or retracted consent during the study term.

The methodology was implemented in four consecutive steps. The initial part involved doing a baseline evaluation and traditional SDL. Students completed a 10-item pretest to assess their foundational knowledge of microbiology and their understanding of AI literacy. After the pretest, they were tasked with Assignment 1, which necessitated the development of a thorough learning module utilizing solely conventional resources, including textbooks and regular web searches, but eschewing AI techniques.

The second phase comprised an intervention consisting of a structured one-hour training session on AI-assisted learning. This course familiarized students with a systematic prompting approach aimed at improving the precision of AI-generated content and reducing inaccuracies. The workflow encompassed establishing a distinct persona and objective for AI (for instance, directing it to operate as a medical educator), supplying contextual prompts consistent with pertinent guidelines such as Indian medical standards, and progressively refining outputs into organized formats with headings such as "principle," "clinical application," and "limitations." An essential aspect of this training was the verification protocol, necessitating that students cross-verify all AI-generated facts and references against authoritative textbooks by Ananthanarayan and Apurba Sastry.

During the third phase, students completed Assignment 2 utilizing AI-assisted SDL. They were permitted to utilize sanctioned AI technologies, chiefly ChatGPT and Gemini, while rigorously following the sequential prompting workflow and verification protocol instructed throughout the training session. This phase allowed students to implement AI-assisted learning methodologies in a systematic and organized way.

The fourth phase concentrated on evaluation and feedback. Upon completing both assignments, students undertook a post-test, including multiple-choice questions, to assess their learning outcomes. Furthermore, they filled out a comprehensive feedback questionnaire to document their experiences, perceptions, and confidence regarding AI-assisted learning.

The assessment rubric was developed after reviewing published literature on educational assessment and CBME. The rubric consisted of 10 domains: correctness, relevance, completeness, organization, clarity, use of examples, inclusion of visuals, critical thinking, appropriateness, and originality. Each domain received a score ranging from 0 to 2, culminating in a maximum aggregate score of 20. Before implementation, the rubric was subjected to content validation by two independent subject matter experts to confirm its consistency with MBBS curriculum objectives and to establish face validity. Two evaluators employed this validated rubric to assess each assignment individually. Prior to formal assessment, the rubric was pilot-tested on five sample assignments to ensure clarity and consistency among evaluators.

In addition to rubric-based evaluations, students maintained time logs to record the total time spent on each task, enabling a comparison of efficiency between traditional and AI-assisted SDL. Knowledge acquisition was evaluated by pre- and post-tests of 10 multiple-choice questions, facilitating the measurement of cognitive enhancement throughout the study.

The analysis of the data used Python-based statistical tools, such as Pandas. Paired t-tests were utilized to compare the mean rubric scores, knowledge test scores, and time-to-completion between the traditional and AI-assisted phases. The intervention's impact size was determined utilizing Cohen’s d. The inter-rater reliability between the two independent evaluators was evaluated using the intraclass correlation coefficient (ICC). Furthermore, descriptive statistics, including frequencies and percentages, were used to analyze the feedback data.

Participants were not randomized to study groups because the objective of this educational intervention was to evaluate the effect of structured AI-assisted SDL within the same cohort. Therefore, each participant served as his or her own control by completing both conventional SDL and AI-assisted SDL sequentially. A concurrent control group was not included because all students were expected to receive the educational intervention as part of the study protocol.

The research was performed in compliance with institutional ethical standards. Participation was voluntary, and signed informed consent was secured from all students before inclusion. To maintain secrecy and privacy, all gathered data were anonymized. The use of AI techniques was limited to the development of academic content under faculty oversight, with no involvement of patient-related data at any point during the project.

## Results

Based on the analysis of the study data provided in the evaluation and feedback files, the following tables present the domain-wise comparison and inferential statistics for the project.

Domain-wise comparison of traditional vs. AI-assisted outcomes

The analysis comparing AI-assisted SDL to traditional methods indicates a notable enhancement in student performance. Assignments supported by AI consistently received higher mean scores across all rubric domains, with improvements ranging from approximately 39% to over 70%. The areas of organization, completeness, and clarity saw the most significant advancements, with AI-generated responses proving more comprehensive, well-structured, and easier to comprehend. Furthermore, there was a substantial increase in both accuracy and relevancy, demonstrating that AI-assisted content was not only precise but also relevant to the subject matter. Table 1 compares the mean scores (out of 2.0) for each specific domain of the assessment rubric across the two study conditions.

**Table 1:** Domain-wise comparison of traditional vs. AI-assisted outcomes.

Rubric domain	Traditional mean (SD)	AI-assisted mean (SD)	% Improvement	t-value	p-value	Cohen’s d	Effect interpretation
Accuracy	1.34 (0.48)	1.95 (0.22)	+45.5%	10.85	< 0.001	1.65	Large: AI content was significantly more accurate.
Relevance	1.38 (0.49)	1.92 (0.27)	+39.1%	9.14	< 0.001	1.37	Large: AI ensured content was strictly on-topic.
Completeness	1.15 (0.36)	1.88 (0.33)	+63.5%	13.55	< 0.001	2.11	Huge: AI covered all aspects of the prompts.
Organization	1.08 (0.27)	1.85 (0.36)	+71.3%	15.68	< 0.001	2.42	Huge: Structure (headings/bullets) was far superior.
Clarity	1.12 (0.33)	1.82 (0.39)	+62.5%	12.42	< 0.001	1.93	Huge: Language was more concise and understandable.
Clinical examples	1.10 (0.30)	1.76 (0.43)	+60.0%	11.45	< 0.001	1.78	Large: AI consistently provided relevant clinical examples.
Visuals/graphics	0.82 (0.39)	1.35 (0.48)	+64.6%	7.85	< 0.001	1.21	Large: Better suggestions for diagrams/tables.
Critical thinking	0.95 (0.22)	1.55 (0.50)	+63.2%	10.12	< 0.001	1.54	Large: AI aided in synthesizing complex arguments.
Appropriateness	1.22 (0.42)	1.90 (0.30)	+55.7%	11.95	< 0.001	1.86	Large: Content was better suited for the MBBS level.
Originality	0.92 (0.27)	1.48 (0.50)	+60.9%	9.18	< 0.001	1.38	Large: Unexpectedly, AI prompted novel structuring of ideas.

Table of inferential statistics

Table 2 summarizes the key statistical findings, including performance gains, knowledge acquisition, and time efficiency. All domains exhibited substantial to very large effect sizes (Cohen's d ranging from 1.21 to 2.42) and demonstrated highly significant results (p < 0.001). This indicates that the observed changes were not due to chance but rather had a meaningful educational impact. Notably, "huge" effect sizes were found in areas such as organization and completeness, suggesting that AI techniques significantly enhance the quality of academic writing.

**Table 2:** Table of inferential statistics.

Outcome parameter	Traditional/Pre-test mean	AI-assisted/Post-test mean	Difference	p-value (Paired t-test)	Cohen’s d (Effect size)
Rubric percentage score	57.1%	86.2%	+29.1%	< 0.001	2.8 (Large)
Knowledge score (Max 10)	5.2	8.8	+3.6	< 0.001	2.5 (Large)
Time spent (minutes)	44.5 min	25.4 min	-19.1 min	< 0.001	1.5 (Large)

Descriptive statistics for AI verification and confidence

Reliability analysis (ICC = 0.94) revealed excellent agreement between evaluators, demonstrating the consistency and dependability of the assessment procedure. Furthermore, user responses indicated a high level of acceptance of AI tools: most participants preferred using ChatGPT, a significant percentage cross-checked material with textbooks, and most reported feeling more confident and synthesizing information more quickly. Nonetheless, participants encountered certain difficulties, specifically with accuracy and the problem of crafting effective prompts (Table 3).

**Table 3:** Descriptive statistics for AI verification and confidence. ICC: intraclass correlation coefficient.

Metric	Descriptive result
Inter-rater reliability (ICC)	Rater 1 - Mean score: 14.2 (Combined average of both assignments). Rater 2 - Mean score: 14.6. Correlation (r): 0.94. Interpretation: An ICC > 0.90 indicates excellent reliability between the two examiners, validating the rubric's consistency.
AI tool preference	71 (85%) used ChatGPT (GPT-5.2/5.1)
Verification rate	71 (85%) cross-checked AI facts with standard textbooks
Confidence score	65 (78%) felt "More Confident" in creating self-learning content
Synthesize speed	76 (92%) agreed AI helped synthesize info faster than traditional methods
Top challenge	54 (65%) cited "Accuracy concerns" or "Difficulty in prompting"

## Discussion

The results of this study provide compelling evidence that systematically integrating generative AI into SDL significantly enhances medical students' academic performance and the efficiency of their learning processes. To distinguish between "linguistic fluency," a strength of AI, and "medical accuracy," which remains the responsibility of the learner, it was essential to employ a purpose-built, internally validated rubric instead of a generic writing assessment [10]. Our tailored rubric facilitated a comprehensive evaluation, enabling us to assess "Accuracy" and "Critical Thinking" separately from "Organization" and "Clarity." In contrast, generic tools may conflate polished grammar with actual content knowledge. This differentiation was critical in avoiding the risk of potential inaccuracies in factual content or clinical relevance being masked by the "halo effect" of AI's superior formatting [11].

An important methodological strength of this study is the standardized intervention protocol, which enhances reproducibility. The AI training session, prompting workflow, verification procedure, assessment rubric, and statistical analysis plan were predefined and consistently applied to all participants. Furthermore, the within-subject design minimized confounding caused by differences in baseline academic ability because each student acted as his or her own control.

Cognitive scaffolding and performance enhancement

The most notable outcome was the 29.1% rise in mean rubric scores, which went from a traditional mean of 57.1% to an AI-assisted mean of 86.2%. This improvement was constant across all 10 rubric domains rather than just one. In particular, the "Huge" impact sizes seen in "clarity" (d = 1.93) and "organization" (d = 2.42) indicate that AI functions as a potent cognitive scaffold. AI enables students to convey knowledge in a more logical and comprehensible style by handling the structural complexity of medical topics, such as the MALDI-TOF (matrix-assisted laser desorption ionization-time of flight) procedure. AI detractors frequently claim that these tools promote "surface learning" or intellectual sloth [12]. Critical thinking scores, however, significantly improved (d = 1.54), according to our findings. This implies that students have more cognitive capacity to concentrate on synthesizing clinical applications and comprehending the limitations of the technology when they are released from the mechanical strain of "information hunting" [13].

Time management and efficiency

In the context of the demanding MBBS curriculum, the completion time reduction from 44.5 minutes to 25.4 minutes (a 43% decrease) is crucial. The amount of material that medical students need to learn often overwhelms them. AI-driven efficiency enables students to achieve high-quality SDL outcomes in less time, which they can then use for interactive peer discussions or clinical bedside instruction. This finding is consistent with the "time-releasing" capability of AI that has been discussed in recent work on medical education [14].

AI literacy and the "Human-in-the-Loop"

The 85% verification rate that students reported was a crucial part of this study. The majority of pupils felt the need to double-check facts with their textbooks, even if the AI output was of high quality. This "healthy skepticism" is the basis of AI literacy. The fact that 65% of students mentioned accuracy issues as a problem suggests that they saw the AI as a drafting helper that needed human supervision rather than as an unfailing oracle. The improvement in scores was objective and not the product of grader bias, as confirmed by the high inter-rater reliability (ICC = 0.92). It supports the application of a defined rubric for assessing medical information produced by AI [15]. Inter-rater reliability analysis was used throughout the study to further assess the validated tool's dependability. An ICC of 0.92 was obtained by two separate raters using the rubric to evaluate the assignments, demonstrating excellent agreement and consistency in the usage of the assessment method.

Limitations

Several limitations should be acknowledged. First, the study was a single-center educational study involving one MBBS cohort, which limits external validity. Second, the study employed a pre-post within-subject design without randomization or a concurrent control group; therefore, causal relationships should be interpreted cautiously because learning effects cannot be completely excluded. Third, outcomes were limited to immediate learning performance, and long-term knowledge retention was not evaluated. Finally, institutional teaching practices and prior familiarity with digital learning tools may have influenced student performance.

## Conclusions

Medical pedagogy has evolved significantly with the integration of generative AI into SDL. This study suggests that students can produce better learning content in much less time than with traditional methods, provided they are given an organized "Stepwise Prompting Workflow" and a strict verification process. We conclude that AI should be viewed as a "co-pilot" that enhances the SDL process rather than as a threat to traditional medical education. However, for AI to be effectively utilized, curriculum design must fundamentally shift from teaching students what to look for to teaching them how to validate and ethically use AI-generated content. It is now essential to formally include "AI Literacy" and "Verification Competency" in the MBBS curriculum to prepare future doctors for the digital era.
